# The challenging journey from trauma to post‐traumatic growth: Lived experiences of facilitating and hindering factors

**DOI:** 10.1111/scs.13037

**Published:** 2021-10-28

**Authors:** Hulda S. Bryngeirsdottir, Sigridur Halldorsdottir

**Affiliations:** ^1^ School of Health Sciences University of Akureyri Akureyri Iceland

**Keywords:** post‐traumatic growth, psychosocial nursing, public health, phenomenology, qualitative approaches, qualitative interviews, rehabilitation, trauma

## Abstract

**Background:**

Many people experience psychological trauma during their lifetime, often negatively affecting their mental and physical health. Post‐traumatic growth is a positive psychological change that may occur in an individual after having processed and coped with trauma. This journey, however, has not been studied enough.

**Aim:**

The purpose of this phenomenological study was to explore people's experience of suffering psychological trauma, the personal effects of the trauma and the transition from trauma to post‐traumatic growth.

**Methods:**

A purposeful sample of seven women and five men, aged 34–52, were selected whose backgrounds and history of trauma varied, but who had all experienced post‐traumatic growth. One to two interviews were conducted with each one, in all 14 interviews.

**Results:**

This study introduces a unique mapping of the challenging journey from trauma to post‐traumatic growth through lived experiences of people who have experienced trauma as well as post‐traumatic growth. Participants had different trauma experience, but their suffering shaped them all as persons and influenced their wellbeing, health and view of life. Participants described post‐traumatic growth as a journey, rather than a destination. There was a prologue to their journey which some described as a hindering factor while others felt it was a good preparation for post‐traumatic growth, i.e. to overcome difficulties at an early age. Participants described six main influencing factors on their post‐traumatic growth, both facilitating and hindering ones. They also described the positive personal changes they had undergone when experiencing post‐traumatic growth even though the epilogue also included heavy days. A new theoretical definition of post‐traumatic growth was constructed from the findings.

**Conclusion:**

The results suggest that the journey to post‐traumatic growth includes a recovery process and certain influencing factors that must be considered. This information has implications for professionals treating and supporting people who have suffered traumas.

## INTRODUCTION

Many people suffer psychological trauma during their lifetime. Suffering trauma can play a large role in the development of various psychological problems [1, 2, 3, 4, 5]. Trauma occurs due to a threatening and unexpected event that the individual does not control and may have challenged the perception of living in a safe and predictable world [6, 7, 8]. The more the event affects the person directly, the greater the risk of emotional harm [5, 9, 10].

Known negative emotions following trauma are shame, sadness or depression, anxiety and guilt, especially after a personal trauma [2, 11, 12]. Research has shown that a variety of risk and protective factors affect an individual's response to personal trauma, such as the person's previous suffering of trauma, the severity of the trauma and other's reaction to the trauma [13]. Social support is a positive, protective factor [14, 15].

Various factors other than the trauma itself may affect people's reactions to trauma, e.g. stress levels before the trauma [8, 16] and current stress [17]. Furthermore, relief, adaptability, what the person considers important, life‐satisfaction and positivity are also key factors when it comes to individual response to trauma [17]. Research indicates that those who suffer more than one type of trauma may be more likely to experience health problems than those who suffer a single trauma. However, other factors such as the severity of the trauma [7, 18], culture, and type and combination of traumas are also important in this regard [4].

Trauma causes stress and stressful events in people's lives can have a measurable effect on their neurological and immune responses which can affect the individual's mental and physical health, well‐being and quality of life [19, 20, 21, 22, 23]. Excessive stress interferes with the coordination of the body's defence systems, which can then adversely affect an individual's physical and mental well‐being. If the stress is prolonged, it can contribute to long‐term negative effects on the individual [24, 25].

Experiencing potentially harmful life events can lead to feelings of helplessness or great fear, causing traumatic stress and even post‐traumatic stress disorder (PTSD) [8, 15, 26] which is one of the most serious and inhibiting types of stress [5, 27]. PTSD has a negative effect on the individual's physical and mental health; its main symptoms are intrusive thoughts about the trauma, avoidance of what is reminiscent of the trauma, hypersensitivity [2, 28, 29] and fear [2, 6, 30]. Cumulative stress due to previous trauma can affect whether, how much and for how long people suffer from PTSD [7, 26, 31]. Studies indicate that experiencing safety and social support reduces the likelihood of developing symptoms of PTSD [6, 15].

Post‐traumatic growth is a positive psychological change that may occur in an individual who has suffered a trauma. Research suggests that such growth consists of five main factors: people experience increased spiritual development, see new possibilities in life, value life more than before, experience increased personal strength and better relationships with others [32, 33]. When assessing post‐traumatic growth, all these factors are considered [34]. Research has shown that some of those who have suffered trauma describe these extensive positive changes in their lives as a result [35, 36]. Individuals living in tornado areas in Australia were studied by Pooley et al. [10] regarding stress and post‐traumatic growth. The results of that study show that those who have resilience and self‐efficacy are more likely to positively grow despite experiencing serious threats or adversity. Thus, trauma can sometimes increase people's ability to cope, adapt and even embrace a new stressful reality so that they become stronger than ever before [7]. Seeing post‐traumatic growth as a goal, the journey from suffering a psychological trauma towards post‐traumatic growth needs to be better understood so that professionals can better guide them on their journey. Therefore, the aim of the study was to increase knowledge and deepen understanding of the key influencing factors in suffering a psychological trauma, the personal effects of experiencing the trauma, and how people describe the transition from trauma to post‐traumatic growth.

The research questions were:

1. What is people's experience of suffering psychological trauma?
2. What is people's experience of mental and physical symptoms following trauma?
3. What is people's experience of factors affecting their journey from trauma to post‐traumatic growth?
4. What is people's experience of post‐traumatic growth?
5. What is the epilogue of people experiencing trauma and post‐traumatic growth?
6. Based on the participants' experience what is the theoretical definition of post‐traumatic growth?

## METHODOLOGY

To answer the research questions, a phenomenological method was used, i.e. the Vancouver‐School of doing Phenomenology (in short Vancouver‐School), which aims to understand participants’ experiences of certain phenomena by examining their description and interpretation of their experiences [37]. Each participant is seen as a case study, and the method is based on analysis of individual cases (steps 1–7) and then an inter‐cases analysis (steps 8–12). The implementation of the study was conducted consistent with these 12 main research steps (see Table 1).

**TABLE 1:** The 12 basic steps of the research process of the Vancouver School in this study

Steps in the research process	What was done in the present study
Step 1. Selecting dialogue partners (*the sample*)	Fourteen participants that self‐reported post‐traumatic growth (PTG) participated in the study. When interviewed it was revealed that two of them did not meet the criteria of PTG, so their data were not considered in the results. Participants were therefore twelve, seven women and five men
Step 2 Silence (*before entering a dialogue*)	The researchers reflected upon their preconceived ideas and consciously put them aside as much as possible
Step 3 Participating in a dialogue (*data collection*)	One interview was conducted with each of the twelve participants, two interviews with two participants, in total 14 interviews. The first author conducted all the interviews which were recorded, written verbatim in a computer, and encrypted
Step 4. Sharpened awareness of words (*data analysis*)	Data collection and data analysis ran concurrently. The interviews were read repeatedly by the researchers and comments were written on the margins to find the core of the interview and trying to answer the research question
Step 5. Beginning consideration of essences (*coding*)	Each interview was analysed in detail, and main themes and subthemes were constructed
Step 6. Constructing the essential structure of the phenomenon from each case (*individual case construction*)	The main themes and subthemes in each participant´s story was highlighted and the most important themes constructed into an individual analytic framework
Step 7. Verifying each case construction with the relevant participant (*verification 1*)	All participants confirmed their individual analytic framework
Step 8. Constructing the essential structure of the phenomenon from all the cases (*meta*‐*synthesis of all the different case constructions*)	All individual analytic frameworks were compared and constructed into one main analytic framework (Figure 2)
Step 9. Comparing the essential structure of the phenomenon with the data for verification (*verification 2*)	For verification, all the transcripts were read over again and compared to the final analytic framework
Step 10. Identifying the overriding theme which describes the phenomenon (*construction of the main theme*)	The challenging journey from trauma to post‐traumatic growth: Lived experiences of facilitating and hindering factors
Step 11. Verifying the essential structure with the research participants (*verification 3*)	All participants verified the final analytic framework and the main theme, except one participant who did not reply
Step 12. Writing up the findings (*multivoiced reconstruction*)	The participants are quoted directly to increase the trustworthiness of the findings and conclusions

**FIGURE 1:**
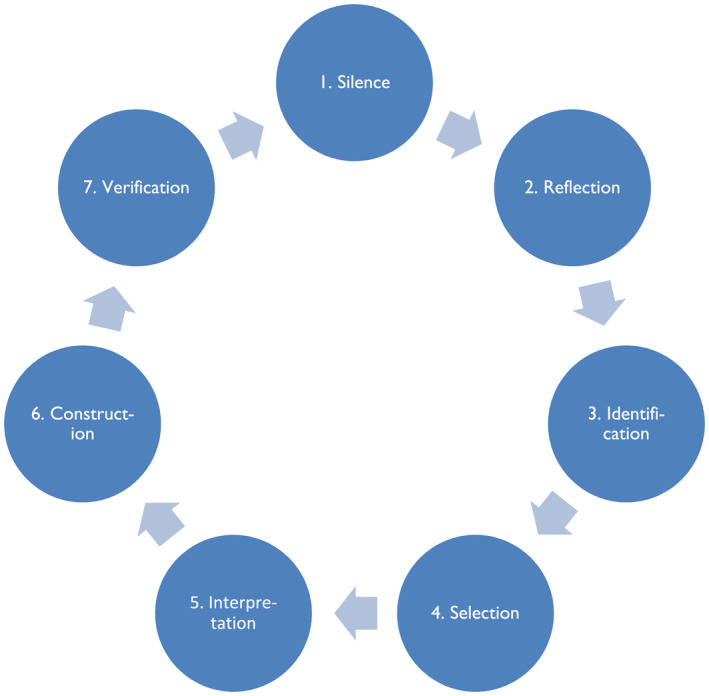
The research process of doing phenomenology in the Vancouver School [Modified figure from Halldorsdottir, S. (2000)[37, p. 56]. Used with permission]. This cycle is repeated in every of the 12 steps of the Vancouver School

The research process in the Vancouver School is characterized by seven main cognitive factors which are set up as a circular process which is repeated throughout the research process (see Figure 1).

### Inclusion criteria

Inclusion criteria included having suffered a psychological trauma and having been unable to work or having dealt with reduced working capacity following the trauma due to physical and/or mental health reasons caused by the trauma, followed by a self‐described post‐traumatic growth. Further inclusion criterion was that a participant had not received a psychiatric diagnosis before the psychological trauma and that more than six months had passed since the participants suffered the trauma.

### Sample

The participants were recruited in collaboration with different rehabilitation resources and among colleagues, who introduced the participants to the first author. A theoretical definition of post‐traumatic growth was used when recruiting participants (see Table 2). To get the widest possible scope in the study, emphasis was placed on selecting participants who had suffered different types of traumas. A purposive sample was used, and interviews were conducted with seven women and five men. The participants were aged 34–52 and had all been unable to work or dealt with reduced working capacity following their trauma.

**TABLE 2:** The working definition of post‐traumatic growth (PTG) used in the study

An individual who has reached post‐traumatic growth experiences positive personal changes as a result of a struggle with a traumatic event. The individual has increased personal strength, improved relationships with others, experiences positive changes in attitudes and appreciation towards life and sees new possibilities in life. The experience, though negative in itself, has had positive meaning for the person

Note

[The researchers based their definition of post‐traumatic growth on Ref. 16, 17, 28, 32, 33, 34, 35, 36]

### Data collection and data analysis

An interview guide designed by the researchers was used, and the length of the interviews ranged from 23 to 81 min. The data collection and analysis are described in Table 1. Examples of interview questions are, e.g. how would you describe your experience of suffering a psychological trauma? Can you describe the mental and physical symptoms following the trauma? Can you describe your experience of factors affecting your journey from trauma to post‐traumatic growth? How would you describe your experience of post‐traumatic growth? Can you tell me about the epilogue of your experience of trauma and post‐traumatic growth? The interviews were conducted via a tape recorder in places chosen by the participants during a three‐month period. After the initial data analysis of the first author, both authors were involved in the data analysis and data presentation. The first author is experienced in interviewing people, and the second author has a 34‐year experience of doing qualitative research.

### Validity and reliability

To reduce the likelihood of homogeneity, participants were selected who had suffered different kinds of trauma, of both sexes and of diverse ages. Interviews were conducted until the researchers agreed that data saturation had been achieved and the research questions could be answered.

The first author who conducted and did the primary analysis of all the interviews is an MSc nurse with broad experience of nursing for the past 25 years. She has considerable knowledge and experience of in‐depth interviews and of building rapport with respondents. She has personal experience of suffering psychological trauma followed by post‐traumatic growth. Blythe et al. [38] investigated the challenges of being an insider in storytelling research and found that this can have both advantages and disadvantages. It is important that researchers are aware of these possibilities and take measures to minimise their effects [38, 39]. The first author was aware that her experience of post‐traumatic growth could influence the research process. To minimise that risk, she consciously set aside her preconceived ideas of the subject, using the research process in the Vancouver School (see Figure 1) and by supporting the results by direct quotations from the participants. The research process in the Vancouver‐School has inbuilt verification in steps 7, 9 and 11 which increased the validity and reliability of the study, so all participants reviewed their own individual analytic framework as well as the final analytic framework (all but one who did not respond) (see Table 1).

### Research ethics

The main principles of research ethics guided researchers in the study. The Icelandic National Bioethics Committee granted permission to conduct the study (reference no: VSN‐15‐102). Each participant received an introductory letter and an oral presentation about the study and signed an informed consent. In the introductory letter, possible participants were informed about the purpose of the study, the research method and what was involved in participation. They were informed of their rights to participate voluntarily and to withdraw from the study whenever they wished, as well as of anonymity and absolute confidentiality. Transcripts with anonymised interview data were stored in a locked cabinet in a safe place. All names in the study are pseudonyms.

It was recognised that participation in the study could cause participants’ emotional distress as they were reviewing difficult periods in their lives. Therefore, the first author contacted each participant again by phone 7–10 days after each interview to check on their well‐being and again later in the research process to seek verification of each individual case construction with the relevant participant (*verification 1*, *step 7* in the research process), as well as the verification of the final analytical framework (*verification 3 , step 11*). Participants were offered the psychological support of a mental health professional, free of charge, if they felt the need for one. No participant took advantage of that.

## RESULTS

**FIGURE 2:**
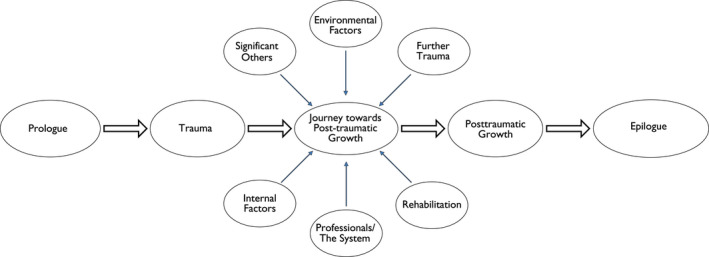
The journey towards post‐traumatic growth

The psychological traumas the participants suffered were negative in themselves and had a lasting effect on their lives. Everyone described how this life experience had shaped them as persons and influenced how they view life today. For the participants, post‐traumatic growth was more of a journey than a destination and there were many influencing factors on the journey, both facilitating and hindering ones. The overall findings are shown in Figure 2, as well as in Tables 3, 4, 5, 6. Figure 2 portrays the six main influencing factors that affected the participant's journey towards post‐traumatic growth. It also shows the prologue to the trauma and, finally, the epilogue, i.e. how the participants felt today, their ‘heavy days’ and the lessons they have learned from their challenging life experience.

### The prologue: The participants' history and past traumas

When participants started to tell the story of their journey from trauma towards post‐traumatic growth, they talked about their previous traumas. They described how previous life experiences influenced their reaction to the latest trauma that almost caused them to lose their health. Ten participants spoke unsolicited about their childhood, describing challenging conditions and poor parenting methods at their childhood homes and the psychological traumas they suffered as children. Hannah reminisced:

[There] was a lot of drinking in the home, lack of money and such. You watched domestic violence and your parents were fighting and you did not get this warmth in childhood as you show your own children today. There was no kiss and "I love you" … You got no help with homework or anything so you were teased throughout primary school and performed very badly in school. I did not understand anything.

Participants linked their characters and defences in adulthood to their childhood experiences, but few of them had processed their childhood suffering and traumas. Gregor recounted:

I was raised in such a way that you should not complain, and you should not cry. I should be big and strong, and I've always been like that … at the end I just broke. There was something [within me] that just broke.

Seven participants felt that difficult experiences in their youth had been a good preparation for the trauma they suffered later in life. Liam explained: ‘I think that when I was very young, I grew some kind of "shell" [by suffering traumas as a young boy] and somehow this "shell" just kept me going in life … but still this "shell" was not comfortable to wear’. However, several participants described how the snowball effect of their previous traumas caused them to ‘lose it.’

### Suffering the psychological trauma

The psychological traumas that participants suffered and caused upheavals in their lives were of various kinds. The traumas were all very personal and were related to the participants’ own life and health or those of their loved ones. All the traumas had serious and very personal consequences for the participants who came close to losing their mental and physical health. The traumas included accidents, serious illness of a child, suicide of a loved one, experiencing violence or severe bullying, losing a job, serious illness, divorce, mental illness of a loved one, adultery, serious financial problems and loss of housing. The period following the trauma was often accompanied by intense internal conflict and discomfort, which initially had a negative effect on the individual's self‐esteem and interactions with others. Six participants described their feelings of anxiety and depression. Seven participants showed strong symptoms of PTSD, and three of them received a formal diagnosis of PTSD. The trauma and the process that followed had profound effects not only on the participants but also on their personal network, especially those closest to them. All the participants described mental and physical symptoms following the trauma (see Table 3), and these symptoms caused a significant reduction in their quality of life.

**TABLE 3:** Mental and physical symptoms of suffering psychological trauma

Mental symptoms	Physical symptoms	Quotations from participants
Mental numbness, anxiety, fear, feeling of rejection, giving up, worries, loss of vision, self‐criticism, mental breakdown, social isolation, social phobia, depression, weeping, self‐pity, denial, decreased self‐esteem, misery, loneliness, listlessness, difficulties in communication, anger, suicidal thoughts, guilt, mood swings, negative behavior toward others, feeling helpless, sorrow, financial worries, shame, loss of short‐term memory, feeling fragile and sensitive, uncertainty, avoiding certain situations and circumstances	Increased heart rate, shortness of breath, tension, sweat, physical sensitivity, pain, physical impairment, feeling of paralysis, drug use, insomnia, loss of appetite, stomach aches, myositis, head ache, restless sleep	The symptoms I experienced can be compared to being a mental burn patient. Everything is painful. All the sensors are so open. You are so open emotionally that the slightest stimulus is going to hurt. Everything is so exaggerated, and you guard yourself as much as you possibly can to defend yourself (Maria) … the heartbeat increases you know, the breath gets shorter and the fear that life is going upside down… and these symptoms of anxiety, you just get so tense and sweaty… sometimes I thought I was having a nervous breaktdown (Anna)

### Key influencing factors on the journey towards post‐traumatic growth

Despite different backgrounds and trauma histories, participants had several things in common when it came to responding to the trauma, processing it and coping with it on their journey to post‐traumatic growth. According to the participants’ descriptions, there were various factors that influenced how life after a trauma developed, how long the symptoms of the trauma lasted and how they recovered from the psychological trauma. Everyone agreed that support had positive effects on the journey towards post‐traumatic growth, while lack of support had negative effects. The six main influencing factors were significant others, environmental factors, further traumas, internal factors, professionals and the system and, finally, rehabilitation. An overview of the key influencing factors on symptoms of psychological trauma and the process to post‐traumatic growth is found in Table 4.

*Significant others* were very important to the participants and had great impact on their journey either positively or negatively. The trauma generally put more strain on loved ones and their home life.

*Environmental factors*. Reactions from other people in the participants’ close environment regarding their traumas were very different. Participants who experienced compassion and support said it was precious, while lack of support resulted in negative and destructive feelings.

*Further traumas* occurred in the lives of all participants on their journey to post‐traumatic growth, which in all cases had negative effects on their well‐being and delayed the process of post‐traumatic growth. Four participants felt that the help they had already received from professionals in dealing with previous traumas was useful when further traumas occurred. Five participants felt that previous traumas helped them to deal with further traumas. Liam described this:

I felt like I was … so used to this [to suffer trauma] … it did not surprise me anymore. It turned into anger or something. But nothing like "why me" or something like that … it all just stopped surprising me.

The most frequent type of further trauma was related to the participants’ poor economy due to prior trauma.

*Internal factors* of all participants had either positive or negative effects on the symptoms of the trauma, the sequence of events that followed and the processing of the trauma. The most important internal factors mentioned were willpower and courage. Good upbringing, self‐examination and previous suffering of traumas were among the factors that the participants felt had positive effects on their journey. On the other hand, they felt that uncertainty, self‐prejudice, co‐dependency and financial worries were strong negative factors that delayed their journey towards post‐traumatic growth.

*Professionals and ‘the system’*. Eleven participants had used professional help and felt that it had helped them very much. The participants sought advice and assistance of various kinds, e.g. from psychologists, trade unions, family counsellors, health professionals, social services, alternative medicine, lawyers and most participants were satisfied with these services. However, they often found information and guidance on possible next steps unsatisfactory and felt that the various systems pointed at each other. As a result, the participants sometimes experienced complete confusion, which had a negative effect on their mental and physical well‐being and recovery process. So, these services had various effects on their well‐being, either in a positive or negative way.

Six participants considered it necessary to make changes to many existing ‘systems’ in society to better care for people suffering from psychological trauma.

When people suffer trauma [it is important] that there is someone there for you within the health care system who will take you by the hand and guide you for the first week or two, to get you started. Someone who calls just to follow up on this. I think it is crucial for people who suffer traumas … There is also a need for flexibility in the labour market for people who experience such a situation (John).

Nine participants found it difficult to pursue their rights and find ways to respond to the challenging situation in which they found themselves as Ivor explained: ‘The only answer I got was "you have no rights here"’.

*Rehabilitation*. Specialised rehabilitation measures were very important to those participants who utilised them. Participants emphasised the importance of early intervention and long‐term management and support. Most participants received their rehabilitation from vocational rehabilitation services. How they got in touch with those services differed greatly as did the time that elapsed from their trauma until they got to know the rehabilitation services, which was from a few months up to seven years. The participants were satisfied in general with the services and the consultants’ work and described the service as a positive turning point in their recovery process and journey to post‐traumatic growth. They underlined the individual's own will and positivity as the key to success in rehabilitation.

It is meaningless to go in there just to pretend to take part in this … and you must take part with a positive mindset (Liam).

Five participants started or continued their studies and found this to have a constructive effect on their lives.

The rehabilitation services supported people in going back to work. Only two participants went back to their previous jobs; one of those had problems with the company in going back to her job. Employment, however, was very important to the participants and had a constructive effect on their lives but six participants reported hindrances when entering the labour market after their recovery.

**TABLE 4:** Participants' experience of factors affecting their journey from trauma to post‐traumatic growth

	Description of the theme	Quotes of participants
1. **Significant others**		
Children	All participants were parents and described the importance of their children as their solid core in life Most of them described feeling of guilt for failing their children, not being able to care for them as usual when dealing with the trauma	After my trauma I drove straight home… my children were there and I held them all in my arms and I remember that I thought…“this is what matters, this is my core“ (Maria) … my children had to take care of themselves even though they were too young for that responsibility… I didn‘t take care of them, didn‘t buy them what they needed… I wasn't aware of what I was doing to them. That is a really difficult experience to face (Karen)
Spouses (current/former)	Five of eight current spouses affected the process of PTG in a positive way All ex‐spouses had a negative affect on the process of PTG Four participants described how new love had a positive affect on their PTG	My wife held the family together, she got me on my feet… she was the rock in our lives at the time (Ivor) She said she was going to ruin me completely… just leave me in a ditch… to get even (Gregor) It was incredibly refreshing… to meet a man who found me pretty and fun and wanted to be with me because I was me. It was good for my self esteem and I became more optimistic and happy (Maria)
Close family	Most of the close family members were supportive i.e. regarding financial support, psychological support, caring and love, help with children and household, help with the social system etc Some described a lack of support from their loved ones which was difficult for them	The support of the family was incredible… that was the greatest help for us… incredible support (John) My dad was not very supportive… he was a little preoccupied with what others would say… gossip and such… his reaction hurt me, I admit that (Maria)
Friends	Eight participants felt supported by their friends Three male participants described their experience of mixed feelings, carelessness, rejection and negative attitude from their friends	You need this warm friendship or the family that stands with you… and also advice (Anna) … that I had started to take medicine because of my mental distress… they thought it was stupid, that I was overreacting… „you are all fine now aren‘t you?“… like it was all over… (John)
2. **Environmental factors**		
Other people	Participants described different experiences of either support or negative reactions from other people. Some experienced people dividing into groups either for them or against them, others felt prejudiced since their condition was concidered mental illness Seven participants hid their feelings and thoughts from other people in order to protect themselves and maintain their dignity	An acquaintance of mine made it clear to me that it was totally ok to get another medical opinion… which I did… and I met a great new doctor (Beatrix) People that hardly knew anything about my situation had strong negative opinions of me and expressed their feelings to me… it was very uncomfortable and increased my stress and loneliness (Anna) When I was around people, I just pretended to be all right. I felt it was the best way to defend myself. I had no interest in whining about anything around people who asked very personal questions and then maybe went and slandered about me or felt sorry for me. So I just held my head up high and smiled.. and if I felt bad.. I just stayed at home (Maria)
Employer and co‐workers	Participants had mixed feelings towards different reactions of employers and co‐workers. None of them felt significant support from their co‐workers and some experienced people dividing into groups either for them or against them Five participants reported a total lack of care, rejection and negative reaction of their co‐workers, which had very bad influence on their wellbeing	It was a bit difficult to come to work again and experience a bit of two camps. Those who… suddenly looked at me as a seriously ill patient and didn't respected me anymore and the others who just supported me and were happy for me and just "cool!". It was only in my workplace where I experienced this dichotomy (Karen) I could hardly join meal times because of pain and no one cared, no one brought me food or anything… there was a man at the workplace who had cancer… he gave me some of his pain killers… there were no medicine or anything there… (Liam)
3. **Further traumas**		
	All participants experienced further trauma in their recovery process and journey towards PTG. The traumatic events were most often related to poor financial state due to the previous trauma. The consequences of poor finances were sometimes serious for the participant and his/her family and could lead to divorce, poverty, loss of housing, etc	…on top of all that, I was without income there for [a few] months… it all went away… we lost the apartment… we could not pay (John)
4. **Internal factors**		
Resilience	All participants reported resilience in their recovery process and journey towards PTG, i.e. ambition, stubborness, believe in themselves, determination, perseverance, accepting and following the advice of others, positivity, cleverness, intolerance towards the situation	You just have to have the will to learn… are you hard core enough? Although it may be buried deep inside, you must find your internal strength and courage. This is a real struggle… you must be very strong (Karen)
Uncertainty	All participants experienced fear and uncertainty about their future and that of their family, which caused them physical and mental distress	I didn‘t know what all this meant for me. Would I ever get another job? Would I loose my house? Would I have to leave town? So many questions came to my mind… complete loss of control of my situation… I just didn‘t know anything and my head was spinning… the uncertainty was sometimes just killing me (Maria)
Self reflection	All participants went through self reflection, either on their own or with the help of professionals. The self reflection resulted in a change of mindset for the better. That's where the PTG really began	I started to work with my feelings in a different way… [the psychologist] was helping me to see what I could do… and what I hadn't done… when I finally gave up and surrendered the healing process could begin (Ellis)
Self prejudice	Ten participants suffered from self prejudice due to their circumstances following the trauma. The time it took them to reconciliate with themselves varied	I was the loser, the disabled one… I always called myself "the Disabled" (Beatrix)
Childhood and upbringing	Eight participants felt that their upbringing and their parents' expectations of them as children, e.g. to be dutiful and responsible, had been helpful in processing the trauma	I am of course brought up in a home where you were taught that you should always be working… doing what you are supposed to do… taking care of yourself and your family and being independent… (Ellis)
Previous experience of traumas	Seven participants believed that previous trauma affected their recovery process and journey towards PTG, either for the better or for the worse	I didn‘t want to be like this… because of my children… I didn‘t want my children to grow up in the same situation as I had grown up in (Hanna)
Financial concerns	Six participants experienced financial concerns that led to anxiety, discomfort and uncertainty about the future, thus being a particularly strong and negative factor on their recovery process and journey towards PTG	I was just destroyed. … I just wanted to deliver the mail or something, just to try to get some money but I couldn't. The mental side went completely (silence for 4 sec) down the drain (Ivor)
Codependency	Five participants described how their codependency had influenced their whole life in a negative way. They neglected themselves and avoided to face their real situation, which influenced their recovery process and journey towards PTG in a negative way	I was so codependent, I had no idea of who I was… years before [the trauma] I didn't do anything for myself… I just didn't excist… I think I've always been like that… my childhood was challenging, I always had to watch my step and be careful… there was always some uncertainty about what was about to happen (Karen)
5. **Professionals/the system**		
Psychotherapy	Eight participants sought psychological help at some point in their recovery process, which they all considered helpful except one participant	Fortunately, I had psychotherapy to deal with my situation… It matters how you process your trauma, it doesn't matter how trauma happens… it really helped to deal with my situation… to talk about it (John)
Family counseling	Five participants sought family counseling which they found useful	I knew that everything was going wrong… I just didn't know what to do and there she [the family counsellor] gave me the permission to say that I wasn't well, I was sick… to admit that I couldn't continue doing what I was supposed to do (Naomi)
Physician	Five participants talked about the involvement of their GP, which they found caring though one participant wished that the GP had paid more attention to his mental well‐being	My GP was so great, just wonderful. She encouraged me and had so much faith in me and I could call her whenever I wanted… this gave me a great feeling of security (Maria) I saw my GP a lot and I had told him multiple times that my psychological status was not good… I wish he had sent me to a psychologist earlier… then maybe I wouldn't have gone down all this slope… and maybe the slope wouldn't have been so long… (Liam)
Unions	Five participants involved their union after their trauma. Two of them found their union supportive, one described the help as random and two participants didn't perceive any support from their union	The secretary [at the union] told me that I should seek for vocational rehabilitation services… that was a good step forward (Ellis) I called my union and they said that they couldn't help me. And then I had no idea what to do next (Ivor)
Hospital care	Four participants described their experience of hospital care. Three of them were satisfied with the service at the hospital while one participant described a very negative experience at the hospital that he hasn't fully recovered from	It was the arrangement from the hospital… that kept me going… and I remember they also said "now you're just not well", when the downslide came, and "now you just go to bed and pull the quilt up over your head if you need to and after a while it's over". That lesson was very important… to allow yourself to be sick sometimes… and it would not be final (Karen)
Alternative medicine	Four participants found alternative medicine or alternative therapy helpful in their PTG process e.g. meditation, massage and healing	My masseur kept me alive… he had such a good effect on my psychological and physical wellbeing… there I could finally find the relaxation that I needed to carry on… I had to find some relaxation (Maria)
Social services	Three participants used social services. Their perception of the service differed from being supportive to being a humiliating experience	From them [social services] I got all kinds of advice and support regarding the children… and financial support because my situations were just terrible, I had nothing to offer my children (Naomi) We went to see them [the social services]… they said that since I had so much income before my trauma they couldn't help us (Igor)
6. **Rehabilitation**		
Education	Five participants went to school/continued their education following their trauma, which promoted their personal and social courage and encouraged them to think outside the box	It was incredibly refreshing to walk into a completely different world, to take on new tasks, meet people on your own terms, learn something new, and meet such a wonderful attitude and demeanor (Maria)
Labour market	The Rehabilitation Services supported their people in going back to work. Only two participants went back to their previous jobs; one of those had problems with the company in going back to her job Six participants experienced a lack of cooperation and flexibility in the labor market when they wanted to go back to work All participants reported that being able to work was a very important factor in their recovery process and journey towards PTG	The labour market needs to be more flexible towards people recovering from trauma… when you move from 100% working capacity to 100% working incapacity… and then suddenly at one point you are expected to go out there and work a full time job… when some paper [certificate of illness] expires (John) There was everywhere the same answer, "no, you are not suitable because you have too much medical history".. that is when you got some answers. Sometimes you did not even get an answer!… It was awful not even getting an answer! (Ellis) To go to work… to be a part of society, a part of life in general… to experience humor, just talking to people about something without it being non‐stop about illness and that something is wrong… just talking to people who are working, just about everyday issues, the weather… it lifted me to another level (Florence)

### The journey towards post‐traumatic growth

All participants said that post‐traumatic growth had taken place due to their own internal need for change. Each one defined their starting point and where they wanted to go. Then and not before, post‐traumatic growth could begin. For many, it was the children and/or immediate family that made them realise the need for change.

I treated my daughter so badly one time … she stopped talking to me … and in losing her, my bottom was reached … Then I started trying to fix things (Liam).

The need for change in life emerged in different ways, for example after seeing a therapist or with the help of a relative or friend or even through studying. For others, it was an internal factor and ambition that became the trigger for change towards post‐traumatic growth.

All participants expressed their views on the preconditions for achieving post‐traumatic growth and the usefulness of external factors such as external assistance, management and follow‐up. However, in general, the participants felt that their personal qualities were most important in that matter.

Look, I was completely broken down, lying completely flat on the ground and I just allowed myself to lie there. The thing is that sometimes you must lie down. After a certain time, you lift your head, you sit up and look around for a while, and then maybe you lie down again, to get a little more energy. When you are ready you sit up, sit for a while to get your bearings before you get up and move on in your life again. No one can tell you to recover and you just recover. You have to feel the desire yourself and the purpose (Maria).

### Post‐traumatic growth

Participants all described their post‐traumatic growth: how they confront their own feelings more freely, have deeper relations to others, experience personal growth, live more wholesome lives, know themselves better and have a stronger self‐image. Participants described numerous other positive outcomes of their post‐traumatic growth. Some had found their vision, the process being their own resurrection. They reported increased social activity, positivity, patience, being more appreciative of themselves and felt like winners in life. They discussed their feelings of economical safety, less stress, freedom, power, and energy. They said they were in love or ready to look for love, consciously nourishing their strength and did not experience any regrets. An overview of their experience is found in Table 5, and the resulting new definition of post‐traumatic growth from the participants’ perspective is found in Table 6.

**TABLE 5:** Participants‘ experience of post‐traumatic growth

Themes	Description of the theme	Quotes of participants
Confronting own feelings	The participants agreed that accepting their feelings and talk about how they really felt was a very important factor in the process to PTG	The worst you can do is to suppress your feelings… especially the negative ones that grow into a big clump inside your throat… preventing you from talking because you would probably start crying if you dared to talk… today I see negative, difficult feeling as tides… I close my eyes and let the feelings flow to me lika a wave in the sea, I feel the pain in my heart, the regret, the sorrow… allowing my tears to flow… after a while those feelings fade again… I can almost hear the sound from this tide of feelings… and I feel better. Little by little this tide of feelings takes shorter time and occurs less often (Maria)
Improved relationships	All participants reported improved communication and deeper emotional connection with their loved ones	This experience (silence for 6 sec) it kind of shows me (silence for 5 sec) how strong the family can be… if it sticks together… we are now stronger together than we were before… (Ivor)
Personal growth	Participants considered themselves as stronger, deeper, better and more mature individuals than before. They enjoyed life in the present and had learned to appreciate and find happiness in “the little things in life“.	I want to see my life and all that has happened to me in a positive way. My experience has made me a better and more understanding person… (Hanna) I'm not running towards the finish line anymore… it's the walk itself that matters, the things I get from each day, the communication with others, my family and friends… not only the fancy house that I am going to buy in 10 years or 5 years… but of course I think about the future (Karen)
Wholesome life	Participants described their increased gratitude, serenity, joy and happiness in life. They emphasized their emotional values more than the material ones, the bright sides in life being more visible than the dark ones though life wasn't perfect. They also underlined the importance of looking forward to the future	… to sit in the garden and feel the warmth from the sun… the aroma from my tasteful coffe in a beautiful cup… to feel the wind and the rain in my face when taking a walk… to receive a small bouquet of crushed flowers from my child… all this filled my heart with joy and gratitude… and I thought: "wow, this is life… thank you" (Maria) I’m always trying to improve myself and grow… I just try to look at the good and positive things today… (Gregor)
Increased self knowledge	Participants became more aware of their mental and physical wellness. They took better care of themselves, set their own limits and handled their circumstances in their own way	Part of the lessons learned is to take small steps… and learn… now I know how many candles it is safe for me to light… I know my limits, I know the circumstances in life that I need to feel good… I know the vehicle [myself] and I just drive the vehicle on the speed it can handle… or is realistic for me (Karen)
Stronger self image	Most participants claimed to be more self‐confident and self‐respectful, being more tolerant of themselves and others. Many of them experienced constant growth	People should stand by themselves, both men and women… life is not easy at all but not daring to do anything is not the easiest way. The traumas occur anyway, whether it is a divorce, death or illness, that's just how it is, we will all be afflicted at some point… (Anna)

**TABLE 6:** A new theoretical definition of post‐traumatic growth based on the participants’ experiences

Post‐traumatic growth can be likened to a personal resurrection in life following psychological trauma. Because of the individual's internal need for change, he or she has managed to process the suffering caused by the trauma. The personal changes experienced include confronting own feelings more freely, consciously nourishing inner strength, having deeper relations to others, experiencing personal growth, living a more wholesome life, and having deeper self‐knowledge as well as a stronger self‐image. Furthermore, the individual enjoys increased social activity, positivity and patience and has feelings of freedom, power and energy, without any regrets. Moreover, the individual feels like a winner in life, is less stressed, more appreciative of own self, others and life in general, seeing new possibilities in life having found a new vision as well as deeper inner peace and wisdom. Even though the negative influences of trauma can be present, the positive factors of post‐traumatic growth are dominant.

### Epilogue: Optimism coupled with heavy days

All the participants said that they were in a good place and that they were optimistic about the future, despite various uncertainties, and intended to continue working on their post‐traumatic growth. Eight participants wanted to share their experiences, which was the reason for their participation in the study, and five of them had already done so. Despite their experience of post‐traumatic growth, participants talked about negative long‐term effects of their trauma, such as ‘heavy days’, impaired work capacity, inner insecurity and other negative outcomes (see Table 7).

**TABLE 7:** Participants‘ negative long‐term effects of trauma in spite of post‐traumatic growth

Themes	Description of the theme	Quotes of participants
Heavy days	Seven participants described their “heavy days“ where they still experience difficult feelings, even years after their trauma. They are conscious of those days and have developed their own personal resources to deal with them. Some participants were still using some medication due to their mental distress following the trauma	I have my "days" in between where I can't stand other people and I just want to turn everything off, but that's just normal you know… it's not like I’m cured in one day… (Florence) I still experience heavy days in between, I don't know if I will get rid of them someday or if they are here to stay but during those days I just let myself be… I know these days will come but I also know that they will pass (Maria) You always fall into a loop [of insecurity] every now and then… but then you just have to know that somewhere there behind, you can spot your strength not only your weakness… you gradually learn to know the symptoms and you stop trying what you have tried multiple times and hasn't worked for you (Ellis)
Impaired capacity to work	Six participants had impaired physical and/or mental work capacity as a result of their trauma. This fact caused them anxiety and uncertainty about the future	These days I have self‐confidence in studying but before I was very confident at the labour market. Now it's time to head to the labour market again… and my physical condition is not so good… how will that end? I've started to worry about that now (Igor)
Inner insecurity	Six participants described their continuing insecurity following their trauma. This fact worried them and made them feel distressed over certain things in their lives	Now I doubt myself much more than I did before… If I take on a job I often doubt that I can complete it and when I complete the job I doubt that my work is good enough… there is some kind of punk in the back of my head that doesn't want to leave me alone… I guess this is my shame or my self‐blame… (John)

Despite the great difficulties they encountered, the participants agreed that the positive factors that accompanied this life experience, the post‐traumatic growth, outweighed the negative factors when they arose. Beatrix explained: ‘I say it and I mean it, you know I'm the luckiest woman in the whole world… because I've been through these traumas which make me the woman I am today’.

## DISCUSSION

This study introduces a unique mapping of the challenging journey from trauma to post‐traumatic growth through lived experiences of people who have experienced trauma as well as post‐traumatic growth. One of the important findings of the study is that according to the participants, post‐traumatic growth is more like a journey than a destination and there are many influencing factors on that journey, both facilitating and hindering ones. The journey included a recovery process and had a prologue, most typically the participants’ negative experiences in childhood e.g., poor home conditions and bad parenting methods. The trauma revolutionised their lives. The consequences of the traumas were serious, and they felt close to losing their physical and mental health. Yet they were thankful for this challenging life experience and described their post‐traumatic growth as a valuable outcome of their suffering. They all described how this life experience had shaped them as persons and influenced their view of life in a positive way even if they still had to deal with ‘heavy days'. A new theoretical definition of post‐traumatic growth, from the participants’ perspective, was constructed from the findings, which is a further contribution to the literature.

The factors that had not been previously identified following a trauma and could therefore be culturally significant for this population is that many participants felt that difficulties in childhood were a good preparation for the traumas they later suffered. This would be worth investigating further. Notably, several participants described how the snowball effect of previous traumas made them almost lose their footing in life.

The participants unanimously agreed that social support positively affected their journey while lacking support had negative effects. This is in line with other research findings [6, 15, 40]. Most of the participants experienced support from friends. However, male participants experienced mixed reactions from friends. Women are known to be more likely than men to respond to traumas with social and nurturing behaviour i.e., tend and befriend, and systematically seek support [30, 41]. This may explain this gender difference to some extent but is worth studying further.

Being able to work and having a job had constructive effects on the participants’ lives. However, some of them experienced lack of both willingness and flexibility on behalf of the labour market when it came to going back to work, as reported in other studies [42, 43].

Many of the participants said that they were not in a condition to seek their rights and find out what options were available to them after the trauma, which made them even more vulnerable. A qualitative study on the experience of individuals dealing with reduced working capacity and their communication with professionals during that period showed that advice and support from caring, professional and humane professionals is valuable [43]. It is important that all professionals, e.g. in the social‐ and healthcare systems, respond to the traumas of their clients through early diagnosis and intervention, as well as support, care and follow‐up.

The participants experienced that they had the willpower and courage needed to reach post‐traumatic growth. It must be borne in mind that only people who had reported post‐traumatic growth were included in the study. It is potentially worthwhile to investigate willpower and courage further among people suffering trauma and even find ways to help them develop these important characteristics.

Participants described improved communication and emotional connection with their loved ones, positive change in self‐image and positive changes in worldview as part of post‐traumatic growth. These descriptions are consistent with Calhoun's and Tedeschi [32] descriptions of the three main factors of post‐traumatic growth.

Many of the participants spoke of the need to make changes to the ‘system’ so that it becomes more flexible and individualized, thus contributing to the person's recovery and post‐traumatic growth. These research findings support the ideas of other studies on how the structure of the system can affect recovery and post‐traumatic growth [44, 45, 46].

### Limitations of the study and future studies

The results of this study describe the influencing factors on the journey to post‐traumatic growth. This information can be useful when treating and supporting people who have suffered traumas. The results are based on 12 participants’ accounts of their suffering of trauma and the experience of post‐traumatic growth. Most participants had attended vocational rehabilitation services, and therefore the sample selection may involve a bias in that regard. Moreover, the participants’ willingness, and ability to express themselves about their experience of trauma and post‐traumatic growth may have influenced the results since many were describing their experience for the first time. It could also be a limitation that participation in this study was based on self‐reported post‐traumatic growth. The psychological traumas that participants had suffered were of various kinds which can be a limitation to this research. Therefore, it would be useful to do further research on post‐traumatic growth among groups of people with defined trauma suffering, such as among women who have survived intimate partner violence.

## CONFLICT OF INTEREST

The authors certify that they have no affiliations with or involvement in any organisation or entity with any financial interest, or non‐financial interest in the subject matter or materials discussed in this manuscript.

## AUTHOR CONTRIBUTION

Study conception/design: HSB and SH; data collection: HSB; data analysis: HSB and SH; drafting of manuscript HSB; critical revisions for important intellectual content SH; supervision: SH.

## ETHICAL APPROVAL

The National Bioethics Committee in Iceland granted permission to conduct the study (reference no: VSN‐15–102).
